# Early detection of poor glycemic control in patients with diabetes mellitus in sub-Saharan Africa: a cohort study in Mozambique

**DOI:** 10.4314/ahs.v22i4.16

**Published:** 2022-12

**Authors:** Fausto Ciccacci, Andrea Manto, Lelio Morviducci, Fabiana Lanti, Noorjehan Majid, Mustafa Agy, Cacilda Massango, Stefano Orlando, Giovanni Guidotti, Maria Cristina Marazzi

**Affiliations:** 1 UniCamillus, Saint Camillus International University of Health Sciences, Rome, Italy; 2 Azienda Sanitaria Locale Roma 1, Rome, Italy; 3 Azienda Sanitaria Locale Roma 1 - Santo Spirito Hospital-Diabetology and Dietology Unit, Rome, Italy; 4 DREAM Program, Community of Sant'Egidio, Maputo, Mozambique; 5 University of Rome Tor Vergata, Department of Biomedicine and Prevention, Rome, Italy; 6 Libera Università Maria Ss. Assunta, Rome, Italy

**Keywords:** Diabetes Mellitus, Non-Communicable Diseases, Health Care

## Abstract

**Introduction:**

WHO estimates 422 million cases of diabetes mellitus worldwide. Mozambique has the second-highest mortality related to DM in the African region.

Objectives of the present study are to provide data about a DM care service in Mozambique and to evaluate early outcomes of treatment.

**Methods:**

The new patients diagnosed with DM in a two-years period in a health centre in Maputo (Mozambique) were included in a retrospective cohort study. Fasting blood glucose (FBG), waist circumference (WC) and BMI were collected at baseline and after three months.

**Results:**

188 patients were enrolled. Median BMI, WC and FBG at baseline were respectively 28 kg/m2(Inter Quartile Range [IQR]23.4–31.8), 98cm (IQR 87–105) and 209mg/dL (IQR 143–295). A non-pharmacological intervention was prescribed for six patients, while 182 patients received metformin 500 mg b.i.d. FBG was significantly reduced at control (226[±103.7]mg/dL vs 186[±93.2]mg/dL, p<0.000); however, glycemic control was reached in 74 patients (39.4%); not controlled patients changed regimen. Elderly patients had a higher glycemic control (adjusted Odds Ratio 2.50, 95% CI 1.11–5.06, p=0.002).

**Conclusion:**

Strategies for early detection of scarce glycemic control are feasible in Mozambique and could lead to prompt regimen switch; an invasive therapeutic approach could be preferable in selected cases to achieve control.

## Introduction

Diabetes mellitus (DM) is a growing health concern worldwide. According to the World Health Organization (WHO), the number of patients with DM increased dramatically in the last decades, reaching a total number of 422 million cases, with about 1.6 million deaths per year^1^. The prevalence of diabetes is rising more rapidly in low- and middle-income countries. Data about DM prevalence in the African continent are incomplete; however, figures are projected to increase up to a 5.35% prevalence in 2035^2^. Many social and economic factors in African societies are leading to a change in lifestyles that could be responsible for this increment^3^.

HIV infection and the rapid spread of antiretroviral treatment, although positive impact on life-expectancy, could be important factors favouring the increment of DM in the continent. HIV infection, that is prevalent in African region, was observed to be associated with increment in diabetes and metabolic syndrome^4^,^5^. Alongside with the infection itself, antiretroviral therapy was found to be associated with increased risk of DM and insulin resistance^6^,^7^.

Moreover, little data are available about DM in Mozambique. The country is experiencing a gradual shift of the epidemiological scenario^8^. A study conducted in 2005 found a 2.9% prevalence of DM and impaired fasting glucose in a representative sample of Mozambican^9^. The Global Burden of Diseases study reports 4,711 diabetes-related deaths in 2019 in Mozambique, the highest rate in the region except for the Republic of South Africa^10^. Improvement in medical care in diabetes in the country is needed, especially for insulin-dependent patients^11^.

Objectives of the present study are: 1) to provide data about a DM care service in Mozambique, 2) to evaluate early outcomes for DM treatment in an African context, and 3) to identify possible challenges.

## Materials and methods

### Study design and setting

A retrospective cohort study was conducted involving patients in care in Zimpeto DREAM centre (Maputo, Mozambique). DREAM program is a public health program run by the Community of Sant'Egidio in several African countries, providing a number of health services, including HIV care, hypertension and diabetes screening and treatment, prevention and early treatment of cervical cancer.^12^–^15^. All the services are delivered according to national guidelines.

### Study population

The present analysis included new patients who accessed the centre in two years of activity (June 2018 – June 2020). Inclusion criteria were: age >30 years, being diagnosed with DM and entering the DM care service in the centre.

### Data collection and definitions

For each patient, routine data about baseline body mass index (BMI), waist circumference (WC), and fasting blood glucose (FBG) at the first access to the centre and at the first control were collected. The first control of BMI, WC, and FBG was scheduled at three months from the treatment initiation.

Diabetes was diagnosed according to national and international guidelines, having FBG level that is equal to or greater than 126 mg/dL^16^.

### Statistical analysis

Statistical data analysis was performed using SPSS 21 software (IBM Corp., Armonk, NY). Variables are presented as median with interquartile range (IQR). Univariable analysis was conducted to investigate factors associated with FBG reduction using a Chi-squared test; a multivariable analysis was used to standardize for the baseline glycemic value. Odds ratios are reported with 95% confidence interval (CI). A paired-sample T-test was used to compare means of BMI, WC and FBG at baseline and control.

### Ethical considerations

The study site is working in collaboration and agreement with the local and national health authorities. All the data included in the analysis were routine data, and patients were anonymized before the extraction. Hence, the study was exempted from specific consent.

## Results

In the period of interest, 188 patients were enrolled. Baseline characteristics of the cohort are shown in table 1. Median BMI, WC and FBG were respectively 28 kg/m2 (Inter Quartile Range [IQR] 23.4–31.8), 98 cm (IQR 87–105) and 209 mg/dL (IQR 143–295). All the patients enrolled in care received specific lifestyle counselling aimed at reducing calorie intake and increasing physical activity. For six patients, only non-pharmacological measures were prescribed. All the 182 patients in treatment started with metformin 500 mg b.i.d. The mean time before the first control was 96 (±55) days.

**Table 1:** Baseline characteristics of the cohort

Gender, n° (%): - Female	147 (78,2)
- Male	41 (21,8)
Median age, years (IQR)	57 (49 – 64)
Median BMI kg/m2, (IQR)*	28,0 (23,4 – 31,8)
Median waist circumference, cm (IQR)**	98 (87 – 105)
Median fasting blood glucose, mg/dL (IQR)	209 (143 – 295)

* n=182

** n=170

The results of the paired sample T-test comparing mean BMI, WC and FBG at baseline and at first control are shown in table 2. All three variables were significantly reduced after follow-up, however only glycemic reduction was considerable enough to have a clinical significance. The mean reduction in FBG was 40 (±110) mg/dL; however, glycemic control (FBG < 140 mg/dL) at first follow-up control was reached only in 74 patients (39.4%). Consequently, for 23 patients' metformin dosage was then increased up to 500 mg t.d.s. or q.d.s.; for 107 patients (58.7%) a second antihyperglycemic agent was added on (Glibenclamide 5 mg o.d. in 84 cases, and Glicazide 80 mg o.d. in 23 cases). Table 3 reports the drug regimens prescribed.

**Table 2:** Baseline and follow-up, paired-sample T-test

		Mean	Standard Deviation (±)	N.	Sign.
Fasting blood glucose, mg/dL	Baseline	226	103.7	188	p < 0.000
	Follow-up	186	93.2	188	
BMI, kg/m2	Baseline	28.6	6.6	170	p < 0.000
	Follow-up	28.5	6.5	170	
Waist circumference, cm	Baseline	98.8	14.1	146	p < 0.000
	Follow-up	97.9	16.9	146	

**Table 3:** Drug regimens prescribed after control

	N.	%
Metformin 500 mg b.i.d.	52	27.7
Metformin 500 mg t.i.d. or q.i.d.	23	12.2
Metformin 500 mg b.i.d. + Glibenclamide 5 mg b.i.d.	84	44.7
Metformin 500 mg b.i.d. + Glicazide 80 mg o.d.	23	12.2
No treatment	6	3.2

Patients who reached a FBG < 140 mg/dL, compared to those how did not, showed lower mean FBG at baseline (184 (±86) mg/dL vs 254 (±104) mg/dL, p<0.000). Regardless of baseline FBG, elderly patients had higher likelihood of having a follow-up FBG < 140 mg/dL (adjusted Odds Ratio 2.50, 95% CI 1.11–5.06, p=0.002).

## Discussion

Our analysis shows the results of a DM care service in Mozambique. Our data show a poor glycemic control after three months of monotherapy with metformin 500 mg b.i.d., with 61% of patient not reaching a FBG < 140 mg/dl after three months of therapy. These results show a similar, or better, metabolic control in comparison with data registered in other African countries; some cross-sectional studies found high rates of poor glycemic control among study participants: 70% in Ghana^17^, 70.8% in Ethiopia^18^, 74% in Cameroon and Republic of Guinea^19^, and 73.52% in Uganda^20^.

Being a cohort study, in our analysis, we had the possibility to early detect poor glycemic control. As a consequence, patients were promptly switched to a more effective therapy. In other studies, in the African continent, poor glycemic control was detected in patients with more longstanding duration of hyperglycemia^17^,^18^. In this perspective, our results emphasize the feasibility of early detection of poor glycemic control in Mozambique.

It's noteworthy, in our court, that metformin, used as starting therapy, leads to a significant reduction in FBG in a short while, even if in a small percentage of patients. According to international guidelines, the daily dose of metformin is about 2,000 mg after a run-in period with lower doses to prevent side effects^21^–^23^. This is in agreement with Mozambican guidelines, which recommends to start with metformin 500 mg o.d., and then to increase to 500 mg b.i.d., in the absence of side effects with a final dose of 2,000 mg per day 24.

Moreover, our results emphasize the role of a more aggressive pharmacological approach to reach earlier the target especially in those patients with concomitant cardiovascular risk factors such as obesity, hypercholesterolemia or hypertension.

Our study has some limits. Having more accurate laboratory follow-up (with multiple blood glucose measurements, or HbA1c) could have provided more insights as well as more comprehensive data about other cardiovascular risk factors could have improved the results. However, our objective was to describe a DM service in Maputo. As it was described in many countries, more detailed laboratory monitoring for DM is not widely available on the field^19^. In this perspective, our results from a real-life setting could provide useful information about the feasibility of early glycemic control.

As many authors suggested, DM care in sub-Saharan Africa needs to be dramatically improved^25^. The early detection of poor glycemic control could be an important key in therapeutic success in patients with DM.

## Conclusion

Our results show that the current approach to DM in African countries has a limited effect, as it results in an effective reduction in FBG, but it fails to reach the target in the majority of patients. In this perspective, strategies for early detection of treatment failure should be implemented and a more aggressive therapeutic approach, especially in patients with higher cardiovascular risk, could be preferable.

• The prudent therapeutic approach based on growing dosages of metformin could have limited effect in reducing fasting blood glucose below the 140 mg/dL threshold.

## Figures and Tables

**Figure 1:**
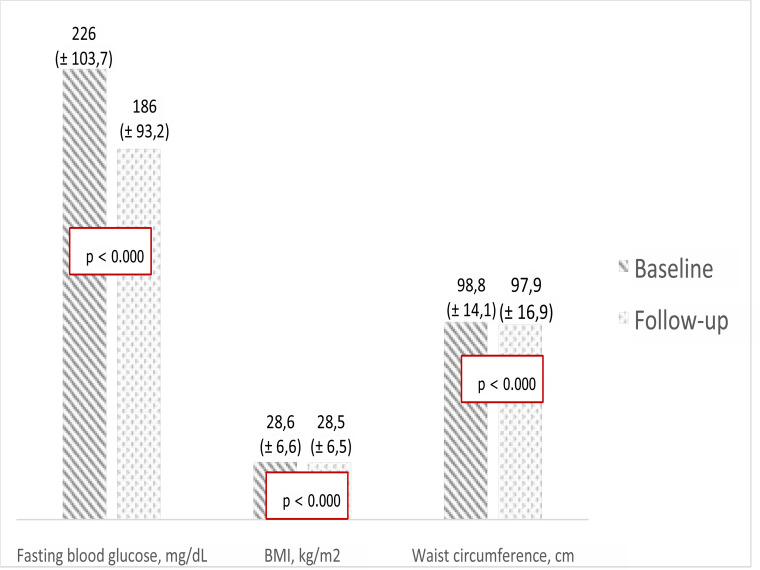
Baseline and follow-up, paired-sample T-test
